# Extracellular vesicles-derived microRNA-222 promotes immune escape via interacting with ATF3 to regulate AKT1 transcription in colorectal cancer

**DOI:** 10.1186/s12885-021-08063-5

**Published:** 2021-04-01

**Authors:** Shiquan Li, Guoqiang Yan, Meng Yue, Lei Wang

**Affiliations:** Department of Colorectal and Anal Surgery, The First Hospital of Jilin University, No. 71, Xinmin Street, Changchun, 130021 Jilin People’s Republic of China

**Keywords:** Mesenchymal stem cells-derived extracellular vesicles, microRNA-222, ATF3, AKT1, Colorectal cancer

## Abstract

**Background:**

Immunotherapy has been recently established as a new direction for the treatment of colorectal cancer (CRC), a gastrointestinal cancer. In this investigation, we aimed to expound how the posttranscriptional regulation modulated by microRNA-222 (miR-222) from mesenchymal stem cells-derived extracellular vesicles (MSC-EVs) affected the AKT pathway and the immune escape in CRC.

**Methods:**

CRC cell malignant phenotype, including proliferation, migration, invasion, and apoptosis, was firstly detected after co-culture with MSC-EVs. miRNAs with differential changes in CRC cells before and after EVs treatment were filtered by microarray analysis. miR-222 was then downregulated to examine its role in CRC cells in response to EVs. Cells were implanted in mice to induce xenograft tumors, and infiltrating T cells was assessed by immunohistochemistry. The mRNA microarray was used to screen target genes, followed by rescue experiments. ChIP and western blot were conducted to validate the downstream biomolecule of ATF3.

**Results:**

After treatment of CRC cells with MSC-EVs, the expression of miR-222 was upregulated, and cell activity was increased. Inhibition of miR-222 decreased CRC malignant aggressiveness in vitro and reduced tumorigenesis and immune escape in vivo. miR-222 targeted and bound to ATF3. Downregulation of ATF3 enhanced CRC cell malignant aggressiveness, tumorigenic capacity and immune escape. Mechanistically, ATF3 inhibited AKT1 transcription and mediated the AKT pathway.

**Conclusion:**

MSC-EVs carry miR-222 to promote CRC cell malignant aggressiveness and immune escape. miR-222 targets and binds to ATF3, which inhibits AKT1 transcriptional activity and thereby mediates the AKT pathway.

**Supplementary Information:**

The online version contains supplementary material available at 10.1186/s12885-021-08063-5.

## Background

Colorectal cancer (CRC) is a very common cancer in the Western World, and in spite of improvements in surgery, chemotherapy and screening, it ranks in the second place regarding cancer-related deaths in this part of the world [1]. The development of CRC occurs in a stepwise manner, developing from a benign preneoplastic lesion to a more metastatic disease that has a poor survival rate (11%), which is motivated by a series of genetic and epigenetic alterations [2]. The escape of immune surveillance of tumor cells is a pitfall that cannot be overlooked, and the aim of immunotherapy is to recruit immune cells and to remove tumor cells by stopping tumor cells from escaping from immune surveillance [3].

Interestingly, tumor- and immune cells-released extracellular vesicles (EVs) exert functional roles in immune processes, involving immune cell priming and activation, and immune escape under both local and systemic contexts [4]. EVs are cells-derived particles ranging from 30 to 1000 nm in size, enclosed within a phospholipid bilayer [5]. EVs from mesenchymal stem cells (MSCs) harbor a healing effect, reverting the malignant phenotype of CRC cells [6]. The roles of EVs between intercellular communication is due to their capability of transferring proteins, lipids and nucleic acids, thus manipulating many physiological and pathological functions in recipient and parent cells [7]. Because of their high abundance and their function as mediators of gene expression, microRNAs (miRNAs), small non-coding RNAs with 19–24 nucleotides, have been identified as potential markers in several cancer types, including CRC [8]. A quantity of EV-miRNAs is linked to development or dismal overall survival of CRC [9]. For instance, the plasma level of miR-30d-5p shuttled by EVs was enhanced in patients with metastatic CRC [10]. Here in our study, miRNA-based microarray revealed that miR-222 was one of the most remarkably upregulated miRNAs in CRC cells co-cultured with MSC-derived EVs. miR-222 is located on the human chromosome Xp11.3 and plays significant parts in the modulation of a broad spectrum of cancers [11]. In the context of CRC, miR-222 overexpression contributed to promoted cell migration and invasion [12]. Additionally, exosomal miR-222-3p was significantly enhanced in patients with lymph node metastasis, indicating its potential predictive roles in papillary thyroid cancer [13]. However, the relevance of miR-222 from MSC-EVs to the progression, especially to the immune escape of CRC remains unclear. Therefore, we examined if miR-222 derived from MSC-EVs is involved in immune evasion in CRC and the potential mechanism of action.

## Methods

### Ethics approval

The study was performed as per the *Declaration of Helsinki* and ratified by the Ethics Committee of The First Hospital of Jilin University. The patient enrolled for MSC extraction signed an informed consent before enrollment. The animal experimental protocol was approved by the Committee on the Ethics of Animal Experiments of The First Hospital of Jilin University. All animal procedures were performed in line with the Guide for the Care and Use of Laboratory Animals issued by the National Institutes of Health (Bethesda, MA, USA).

### Human sample

Fresh tumor tissues were collected from a patient with CRC admitted to The First Hospital of Jilin University on September 13, 2019. The patient was 53 years old and free of a history of other chronic diseases or any other cancers, and had not received radiotherapy or chemotherapy prior to treatment at The First Hospital of Jilin University. The patient was diagnosed with CRC at stage II without lymph node metastasis by colonoscopy and tissue biopsy. We surgically removed the CRC tissues and gave adjuvant chemotherapy to the patient after the surgery. The prognosis of this patient is now good. The excised tissue samples were soaked in 95% ethanol to prevent contamination, and then washed in phosphate-buffered saline (PBS) containing 1% penicillin/streptomycin. The samples were cut into thin slices and detached in type IV collagenase (Thermo Fisher Scientific Inc., Waltham, MA, USA) for 3 h at 37 °C. After detachment, the tissues were rinsed with PBS and passed through a 70 μm sieve (Corning Glass Works, Corning, N.Y., USA). We centrifuged the filtrate and cultured the cells in erythrocyte lysis buffer (Sigma-Aldrich Chemical Company) to eliminate erythrocytes. Cells were grown in low-glucose DMEM (Thermo Fisher) containing 10% fetal bovine serum (FBS) and penicillin/streptomycin at 37 °C, 5% CO_2_ in a cell culture incubator, with the medium renewed every 2 days. The passages were performed at appropriate times. After the fourth passage, MSCs were observed under the microscope for identification and used in subsequent experiments.

### Isolation and characterization of EVs

The cell culture medium was firstly subjected to continuous centrifugation at 300 g for 5 min, at 1200 g for 20 min, and at 10,000 g for 30 min to discard cells and cell debris, followed by a centrifugation at an ultra-high speed of 100,000 g for 60 min at 19 °C using a Sorvall WX Ultra series centrifuge in an F50L-2461.5 rotor (Thermo Fisher). The resulting precipitate was washed with PBS and ultracentrifuged again at 100,000 g for 1 h, and the obtained vesicles were resuspended in Roswell Park Memorial Institute-1640 medium for identification.

EVs were identified by the nanoparticle tracking analysis (NTA) system (NTA 3.2 Dev Build 3.2.16, Malvern Panalytical Ltd., UK). The Brownian motion of the EVs was irradiated by a laser beam and recorded by a camera. NTA was converted by the Stokes-Einstein equation to the size distribution of EVs, which was measured in triplicate. For transmission electron microscopy (TEM), the EVs were fixed with 2% paraformaldehyde and loaded onto carbon-coated copper grids. The grids were placed on 2% gelatin at 37 °C for 20 min and rinsed with 0.15 M glycine in PBS. The morphology of EVs was viewed under a Philips CM120 TEM (Philips Research, Eindhoven, The Netherlands). The expression of EVs-specific markers, tumor susceptibility gene 101 (TSG101) and CD81, was measured by Western blot. Additional file 1 (Supplementary Table S1) presents related antibodies.

### Cell culture and treatment

The cell lines SW480 (CCL-228), HCT116 (CCL-247), 293 T (CRL-3216) were from the American Typical Culture Collection (Manassas, VA, USA). NCM460 cells (MZ-0658) were from Mingzhoubio (Ningbo, Zhejiang, China). To avoid cell contamination, STR genotyping was performed on all cells (including primary MSCs) during the first week of cell culture to confirm cell purity. Mycoplasma contamination was detected in the cells by isolation culture method, and then identified every 2 months during the experiment. The results showed no cross-contamination or mycoplasma contamination in the cells. CRC cells were incubated in complete DMEM containing 10% FBS (Thermo Fisher), 100 mg/mL penicillin and 10 mg/mL streptomycin (Thermo Fisher) for 48 h. The extracted 100 μg/mL EVs were added to the medium and incubated for 24 h. The miR-222 inhibitor, small interfering RNA (si) targeting ATF3 (si-ATF3) and AKT1 (si-AKT1), and their respective negative controls (miR-222 control and NC) were generated by GenePharma (Shanghai, China). The Cy3-labeled miRNA mimic was produced by GE Dharmacon. miR-222 inhibitor, si-ATF3, and si-AKT1 were transfected into EVs-treated CRC cells using Lipofectamine 2000 (Thermo Fisher) according to instructions. Cells were incubated until stable after transient transfection.

### Immunofluorescence staining

After transfection with Cy3-miR-222 mimic, MSCs (1 × 10^6^ cells/well) were co-cultured with CRC cells at a 1:1 ratio using Transwell plates (0.4 mm polycarbonate filter, Corning) for 12 h. CRC cells were placed in the basolateral chamber, and MSCs in the apical chamber. The cells were then fixed with 4% paraformaldehyde at 4 °C for 15 min, incubated with 0.5% Triton-100 X for 20 min, and sealed with anti-fluorescence quenching sealant Vectashield (Vector Laboratories Inc., Burlingame, CA, USA). The presence of Cy3 red fluorescence in CRC cells was observed by fluorescence microscopy (BX63, Olympus Optical Co., Ltd., Tokyo, Japan).

### 5-Ethynyl-2′-deoxyuridine (EdU) labelling

The cells in logarithmic growth period were plated at 1.6 × 10^5^ cells/well and cultivated in 96-well plates for 2 d. EdU (50 mM, Cell-Light EdU Apollo 488 kit, Guangzhou RiboBio Co., Ltd., Guangzhou, Guangdong, China) was supplemented to the cells for a 4-h incubation at 37 °C. The cells were treated with 4% formaldehyde solution for 15 min, with 0.5% Triton X-100 for permeabilization, with 100 mL Apollo Mix for about 30 min at ambient temperature and stained in 100 mL Hoechst33342 staining solution for 30 min before being viewed under a fluorescence microscope (BX63, Olympus). To measure the proportion of EdU-positive cells (red), the EdU-positivity rate was calculated by Image-Pro Plus 6.0 software (Media Cybernetics, Bethesda, MD, USA).

### Microarray analysis

Gene expression analysis of SW480 and HCT116 cells was conducted before and after EV treatment. RNA was isolated from cells using TRIzol reagent (Thermo Fisher) and reversely transcribed into complementary DNA (cDNA) using a Superscript reverse transcriptase kit (Transgene Biotech, Beijing, China). cDNA was hybridized with Human miRNA Expression Microarray V4.0 (Arraystar Inc., Rockville, MD, USA) and GeneChip™ Human Gene 1.0 ST Array (Thermo Fisher). Gene expression data were obtained after incubating the hybridization microarray with DNA in an incubator for 24 h with a GeneChip™ Scanner 3000 7G system (Thermo Fisher), and the resulting data were analyzed by R-project. Affy (Bioconductor) was used for normalization and quality control of expression data, and Pheapmap (Bioconductor) was used to screen differentially expressed genes at |Log_2_FoldChange| > 1, *p* < 0.01 and to plot the heatmap.

### Reverse transcriptase quantitative PCR (RT-qPCR)

After isolation using TRIzol reagent (Thermo Fisher), the RNA was reversely transcribed into cDNA with the help of a Superscript reverse transcriptase kit (Transgene) according to standard instructions. qPCR was conducted on an ABI7300 real-time PCR system (ABI7300, USA) using the Super SYBR Green kit (Transgene). Glyceraldehyde-3-phosphate dehydrogenase (GAPDH) served as an endogenous control for ATF3 and AKT1, and U6 as an endogenous control for miR-222. PCR primers were synthesized by Sangon (Shanghai, China). The specific primer sequences are as follows: miR-222: forward, 5′-CCCTCAGTGGCTCAGTAG-3′, reverse, 5′-CCACCAGAGACCCAGTAG-3′; ATF3: forward, 5′-CTCTGCGCTGGAATCAGTCA-3′, reverse, 5′-CCTCGGCTTTTGTGATGGA-3′; AKT1: forward, 5′-TCCTCCTCAAGAATGATGGCA-3′, reverse, 5′-GTGCGTTCGATGACAGTGGT-3′; U6: forward, 5′-CTCGCTTCGGCAGCACA-3′, reverse, 5′-AACGCTTCACGAATTTGCGT-3′; GAPDH: forward, 5′-AGTGGCAAAGTGGAGATT-3′, reverse: 5′-GTGGAGTCATACTGGAACA-3′.

### Transwell assay

Transwell migration and invasion analyses were performed in 24-well Transwell chambers (0.8 μm pore size, Millipore Corp, Billerica, MA, USA). Transfected CRC cells were trypsinized with 0.25% trypsin and resuspended in FBS-free DMEM. Cells were added to the apical chamber of the Transwell, and DMEM plus 10% FBS was supplemented to the basolateral chamber. Cells were removed after 2 d of incubation. After fixation with 4% paraformaldehyde for 30 min at ambient temperature, cells on the subsurface of the membrane were stained with 0.1% crystal violet overnight. Cells from the bottom of the apical chamber were counted by a microscope (Axiolab 5, Carl Zeiss, Oberkochen, Germany) in four randomly selected areas. For invasion experiments, BD Matrigel (1:8, Corning) was used for the apical chamber coating at a ratio of 30 μL per well.

### Flow cytometry

Apoptosis of CRC cells was evaluated using flow cytometry and the Annexin-V-FLUOS staining kit (Roche Diagnostics, Co., Ltd., Rotkreuz, Switzerland). Cells at the logarithmic growth stage were collected at 48 h post-transfection and resuspended in binding buffer. Apoptotic cells were stained with Annexin V-fluorescein isothiocyanate (FITC)/propidium iodide (PI) for 15 min at ambient temperature with light avoidance. Fluorescence signals were collected by FACSCanto (BD Bioscience, San Jose, CA, USA) and analyzed using FlowJo 8.7.1 software (Ashland, OR, USA).

The surface markers of MSCs were also identified by flow cytometry. The cells were detached with 0.25% trypsin once at an 80% cell confluence, and centrifuged to prepare a single cell suspension. After the cell concentration was adjusted to 1 × 10^6^ cells/mL, 100 μL single cell suspension was reacted with 20 μL human monoclonal antibodies to CD73, CD90, CD105, CD14, CD19, and CD45 (Beyotime Biotechnology Co., Ltd., Shanghai, China) for 30 min, followed by treatment with FITC-labeled secondary antibody (Roche) for 30 min. Fluorescence signals were collected by FACSCanto (BD Bioscience) and analyzed by FlowJo 8.7.1 software.

### Tumor xenografts in nude mice

Thirty 4-week-old female BALB/c nude mice (15 ± 2.52 g) were from Beijing Vital River Laboratory Animal Technology Co., Ltd. (Beijing, China). The transfected CRC cells (miR-222 control, miR-222 inhibitor, miR-222 inhibitor + NC, miR-222 inhibitor + si-ATF3, si-ATF3 + NC, and si-ATF3 + si-AKT1) were injected subcutaneously into mice at 1 × 10^7^ cells per mouse (*n* = 5), and the mouse tumor volume was detected every 7-d using a vernier caliper. Tumor volume changes in nude mice were recorded based on the formula tumor = L × W^2^/2. After 28 d, mice were euthanized by intraperitoneal injection of 1% sodium pentobarbital at 150 mg/kg, and tumors were resected and photographed with weights measured. Following euthanasia, animal death was confirmed by observing the lack of heartbeat, respiratory arrest, pupil dilation, and lack of nerve reflex.

### Immunohistochemistry

Mouse tumor tissues were routinely embedded, de-waxed and hydrated. The tissues were treated with 3% H_2_O_2_ for 15 min at room temperature, followed by another 15-min treatment at room temperature with normal goat serum sealant (Beijing Solabio Life Sciences Co., Ltd., Beijing, China). Afterwards, the tissues were probed with CD3 monoclonal antibody at 4 °C overnight, then with the secondary antibody at 37 °C for 15 min. The diaminobenzidine reaction was carried out by an incubation with 40 μL horseradish-labeled Streptomyces ovalbumin working solution (Solarbio) for 15 min, and then the sections were dehydrated and sealed after a 30-s hematoxylin counter-staining. Microscopy (Axiolab 5, Zeiss) was utilized to observe and count CD3-positive cells in sections. See Additional file 1 (Supplementary Table S1) for antibody information.

### In situ hybridization

The paraffin-embedded tissue sections were dewaxed, hydrated and subjected to a 10-min treatment with 15 mg/mL proteinase K (Exiqon, Denmark) at 37 °C. Double-digoxigenin was utilized to label the miR-222 probe, and tissue sections were immersed in 50 μL hybridization solution and hybridized with the probe (500 ng/mL) at 37 °C for 18 h. The sections were washed with sodium citrate in saline solution, incubated in a sealant containing 2% goat serum (Solarbio) for 4 h at room temperature. Finally, the sections were stained with Nuclear Fast Red (Sangon) for 1 min and visualized by Aperio Scanscope Virtua (Aperio Scanscope FLGL, Aperio) to determine the miR-222 expression.

### 3’untranslated region (3’UTR) luciferase reporter assays

The target genes of miR-222 were predicted using StarBase (http://starbase.sysu.edu.cn/index.php). The fact that ATF3 is a direct target of miR-222 was confirmed using a dual-luciferase reporter gene assay. The wild-type (WT) sequences (GenePharma) with miR-222 binding sites and mutant (MT) sequences in the mRNA 3’UTR were synthesized artificially, and the pmiR-RB-REPORT-ATF3–3’UTR plasmid (RiboBio) was treated with restriction enzymes. The target gene fragments were synthesized and inserted into the pmiR-RB-REPORT vector with miR-222 mimic, respectively. HEK293T cells were collected at 48 h post-transfection, and fluorescence intensity was measured using a luciferase assay kit (Beyotime).

### Chromatin immunoprecipitation (ChIP)

The binding sites between ATF3 to AKT1 were predicted using JASPAR (http://jaspar.binf.ku.dk/). Cells were incubated in 37% formaldehyde at 37 °C for 10 min, collected and added to sodium dodecyl sulfate (SDS) lysis buffer and protease inhibitor complex (Thermo Fisher) for ultrasonic fragmentation. A Pierce Agarose ChIP Kit (Thermo Fisher) was applied. The cells were centrifuged at 10,000 g for 10 min at 4 °C, and reacted with 900 μL ChIP dilution buffer, 20 μL 50x protease inhibitor cocktail and 60 μL ProteinA agarose at 4 °C for 60 min. The mixture was then probed with antibodies at 4 °C, and the precipitated complexes were washed with 60 μL ProteinA agarose and 250 μL eluent. The supernatant was de-crosslinked by adding 20 μL NaCl (5 M) and eluted again by adding 500 μL eluent. After another 1-h incubation with 1 μL RNaseA at 37 °C, DNA fragments were recovered for PCR analysis. Antibody information is exhibited in the Additional file 1 (Supplementary Table S1).

### Western blot

Cells or EVs were homogenized in radio-immunoprecipitation assay lysis buffer (Roche) supplemented with protease inhibitors. Protein concentration measurement was carried out using a bicinchoninic acid protein assay kit (Beyotime). The cultured cells were harvested after an 800-g centrifugation at 4 °C for 5 min, ice-bathed with 5x lysis solution for 10 min, followed by a 10-min centrifugation at 12,000 g and 4 °C. The supernatant was separated by SDS-polyacrylamide gel electrophoresis and trans-blotted to polyvinylidene fluoride membranes (Millipore). The membranes were sealed with 5% skimmed milk powder and probed with primary antibodies for 16 h, followed by the incubation with secondary antibodies for 2 h at 37 °C. See the Additional file 1 (Supplementary Table S1) for all antibody information. Optical density (OD) value measurement was conducted using ImageJ software (version 1.8.0; NIH).

### Statistical analysis

All quantitative results were obtained from triplicate assays and analyzed using GraphPad Prism 6 (GraphPad, San Diego, CA, USA). Data were exhibited as the mean ± standard deviation (SD). The significant difference (*p* < 0.05) between groups was determined by the independent *t* test and one-way or two-way analysis of variance (ANOVA) among multiple groups, along with Tukey’s post hoc test.

## Results

### MSCs-EVs cause a rise in miR-222 expression in CRC cells

**Fig. 1:**
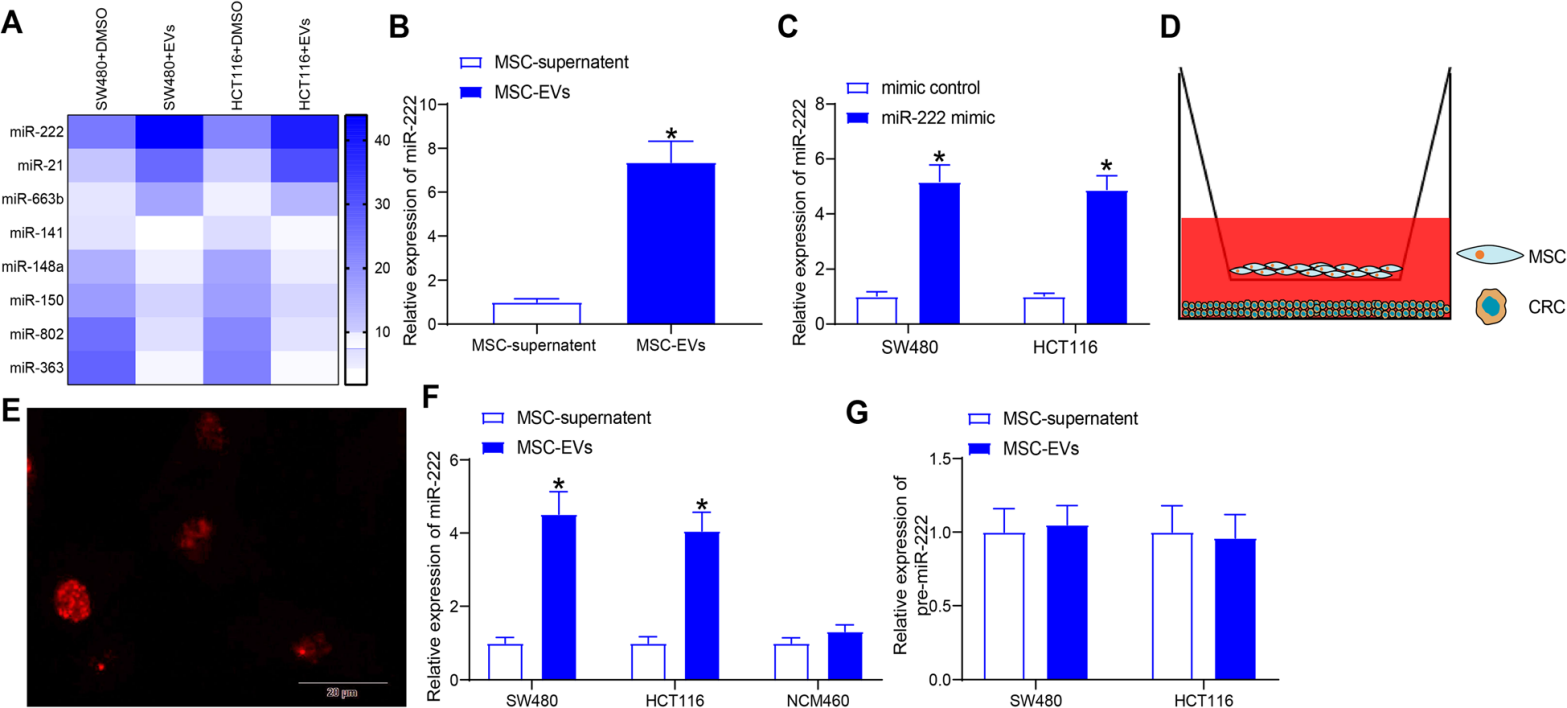
MSC-EVs induce miR-222 expression in CRC cells. **a**, differentially expressed miRNAs after MSC-EVs treatment of SW480 and HCT116 cells using R (Version 3.6.3, ww.r-project.org); **b**, miR-222 expression in MSCs-EVs and in MSCs-supernatant examined by RT-qPCR; **c**, validation of miR-222 mimic transfection by RT-qPCR; **d**, detection of MSC to affect CRC cells via exosomes by Transwell co-culture system; **e**, immunofluorescence detection of cy3-tagged miR-222 mimic fragment in CRC cells; **f**, miR-222 expression in CRC and normal colonic cells detected by RT-qPCR; **g**, pre-miR-222 expression in cells detected by RT-qPCR. Measurement data were presented as mean ± SD. Two-way ANOVA was utilized to analyze data among multiple groups, followed by Tukey’s post hoc test

Elevated cell proliferation was found in CRC cells treated with successfully extracted MSCs-EVs Additional file 2 (Supplementary materials S1 and Supplementary Fig. S1A-F). Microarray analysis of CRC cells before and after EVs treatment was performed to detect differential changes in miRNAs. Cellular RNA was extracted and hybridized with microarrays, and a heatmap was created according to the expression of different miRNAs by Foldchange. In the Fig. 1a, we found that miR-222 was significantly upregulated in EV-treated CRC cells. Examination of miR-222 expression using RT-qPCR revealed a significant increase in miR-222 expression in MSCs-EVs relative to that in MSC-supernatants (Fig. 1b). To investigate whether MSC can act on CRC cells via EVs, we synthesized miR-222 mimic and transfected the fragment with Cy3 fluorescent labeling into MSCs. After determining the successful transfection (Fig. 1c), we co-cultured MSCs with CRC cells using Transwell for 24 h (Fig. 1d). Subsequently, by immunofluorescence staining, we observed a red fluorescent Cy3 marker (Fig. 1e) in the CRC cells of the basolateral chamber, indicating that MSCs did indeed change the expression of miR-222 in CRC cells through EVs. At 24 h post co-culture of SW480 and HCT116 cells with EVs, miR-222 was found to be significantly upregulated in treated CRC cells. In contrast, we found no significant change in miR-222 expression in normal colonic cells before and after MSC-EVs treatment (Fig. 1f). Furthermore, to demonstrate that upregulation of miR-222 was induced by MSC-EVs, we tested the expression of pre-miR-222 before and after EV treatment and found no significant differences (Fig. 1g). This suggests that the upregulation of miR-222 was not a result of endogenous synthesis of miRNAs, but rather the result of direct transfer of MSC-EVs. We tentatively concluded that miR-222 plays an important part in the impact of MSC-EVs on CRC cell activity.

### miR-222 downregulation hampers CRC cell activity

**Fig. 2:**
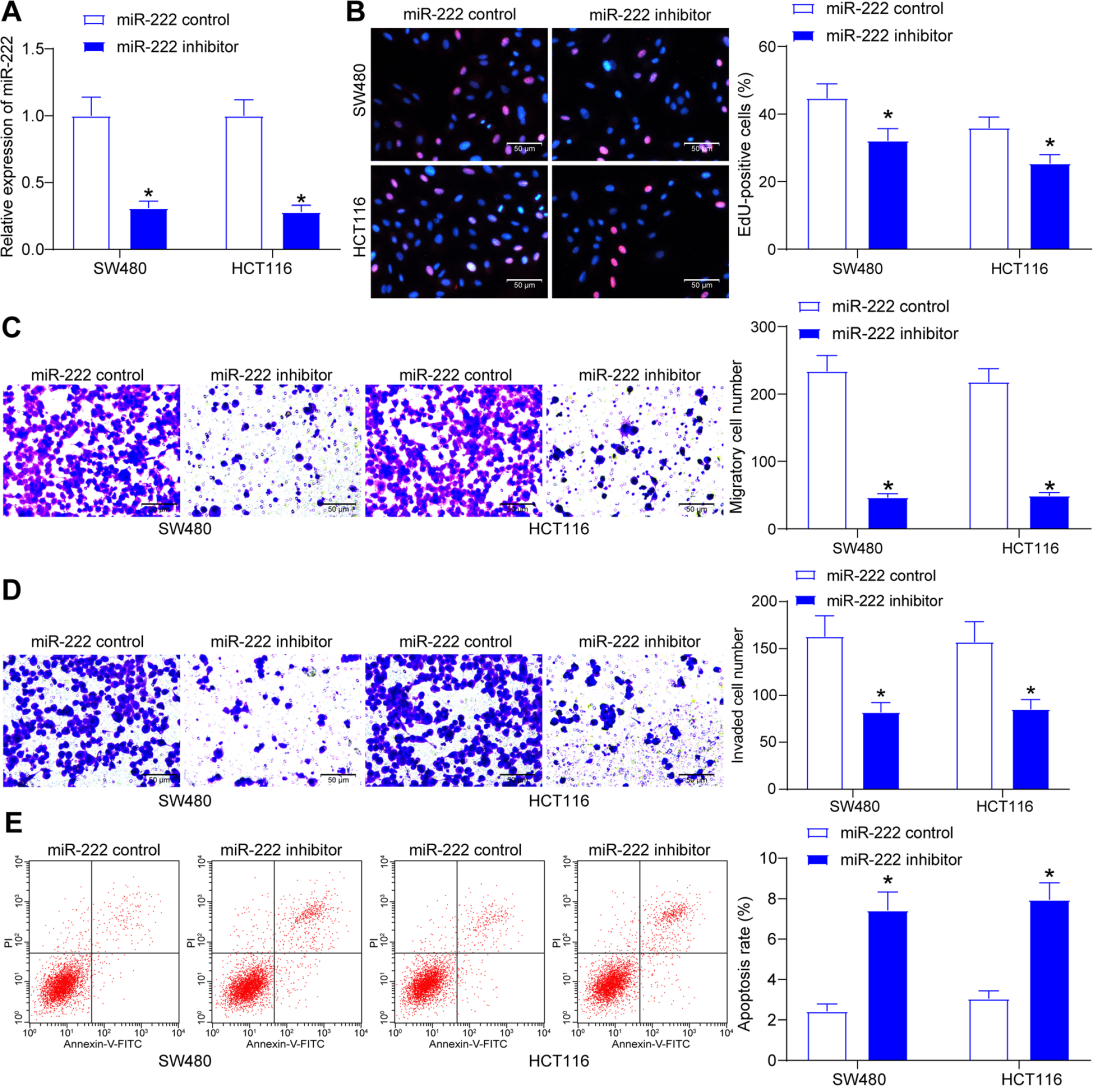
miR-222 inhibitor reduces the CRC cell malignant phenotype induced by MSC-EVs. **a**, the expression of miR-222 in cells determined by RT-qPCR; **b**, cell proliferation activity in cells assessed by EdU assay; **c**, cell migration assessed by Transwell assay; **d**, cell invasion assessed by Transwell assay; **e**, cell apoptosis evaluated by flow cytometry. Measurement data were presented as mean ± SD. Two-way ANOVA was utilized to analyze data among multiple groups, followed by Tukey’s post hoc test

To identify the effect of miR-222 secreted by MSC-EVs on CRC cell activity, miR-222 inhibitor or miR-222 control were transfected into EVs-treated SW480 and HCT116 cells to downregulate miR-222 expression (Fig. 2a). In the aforementioned experiments, we found that EV treatment led to an augment in the proliferative activity of CRC cells, so we first tested the changes in CRC cell proliferative activity after miR-222 downregulation by EdU experiments. Downregulation of miR-222 reversed the effects of EVs and significantly decreased the proportion of EdU-positive cells (Fig. 2b). We then examined changes in cell migration and invasion by Transwell experiments, and microscopic observation of migrated and invaded cells to the basolateral chamber in the Transwell assay revealed that miR-222 downregulation significantly inhibited the pro-migratory (Fig. 2c), and pro-invasive (Fig. 2d) effects of MSCs-EVs on CRC cells. Flow cytometry showed that downregulation of miR-222 significantly mitigated the anti-apoptotic effects of MSCs-EVs and increased both early and late apoptotic cells in SW480 cells and HCT116 cells (Fig. 2e). Through a series of experiments, we observed that miR-222 knockdown reverted CRC cell malignant phenotype caused by MSCs-EVs.

### miR-222 downregulation inhibits immune escape in CRC cells

**Fig. 3:**
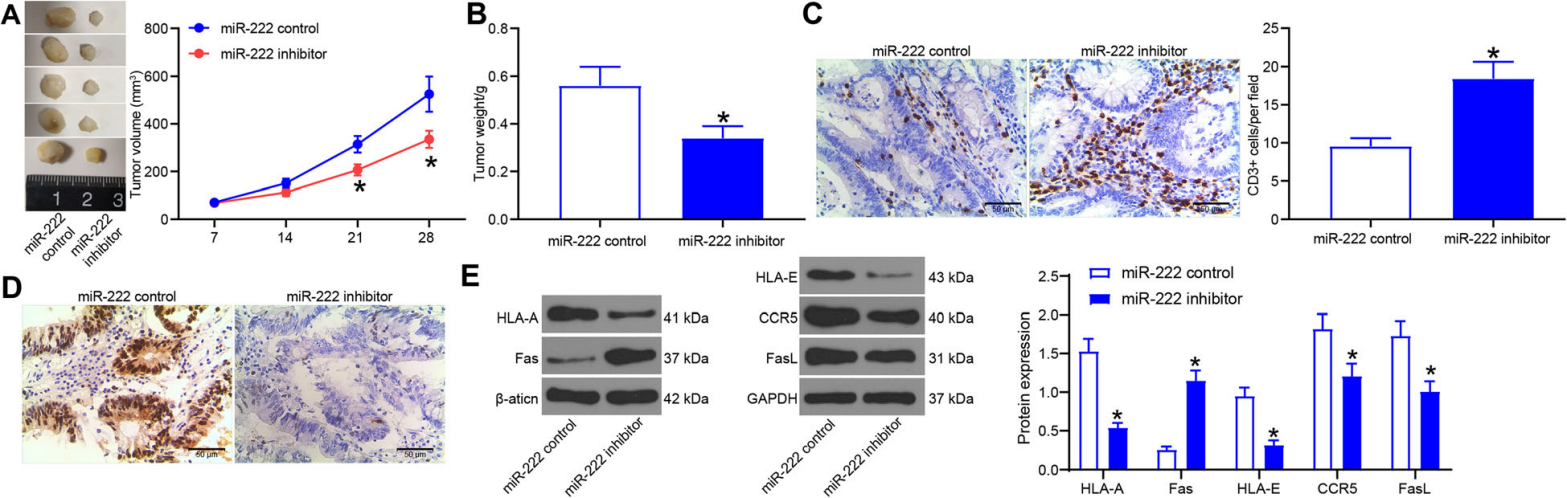
miR-222 inhibitor reduces the CRC tumorigenesis and immune escape induced by MSC-EVs. **a**, tumor volume in xenograft models; **b**, assessment of tumorigenesis by tumor weight; **c**, percentage of CD3^+^ cells in tumor tissues detected by immunohistochemistry; **d**, miR-222 expression in cells by in situ hybridization; **e**, expression of immune escape-related proteins in tumor tissues examined by western blot. Full-length blots are presented in Additional file 3 (Supplementary Fig. S2). Measurement data were presented as mean ± SD. Unpaired *t* test was applied to analyze the differences between two experimental groups (panel **b** and **c**), while two-way ANOVA to analyze data among multiple groups, along with Tukey’s post hoc test (panel **a** and **e**)

**Fig. 4:**
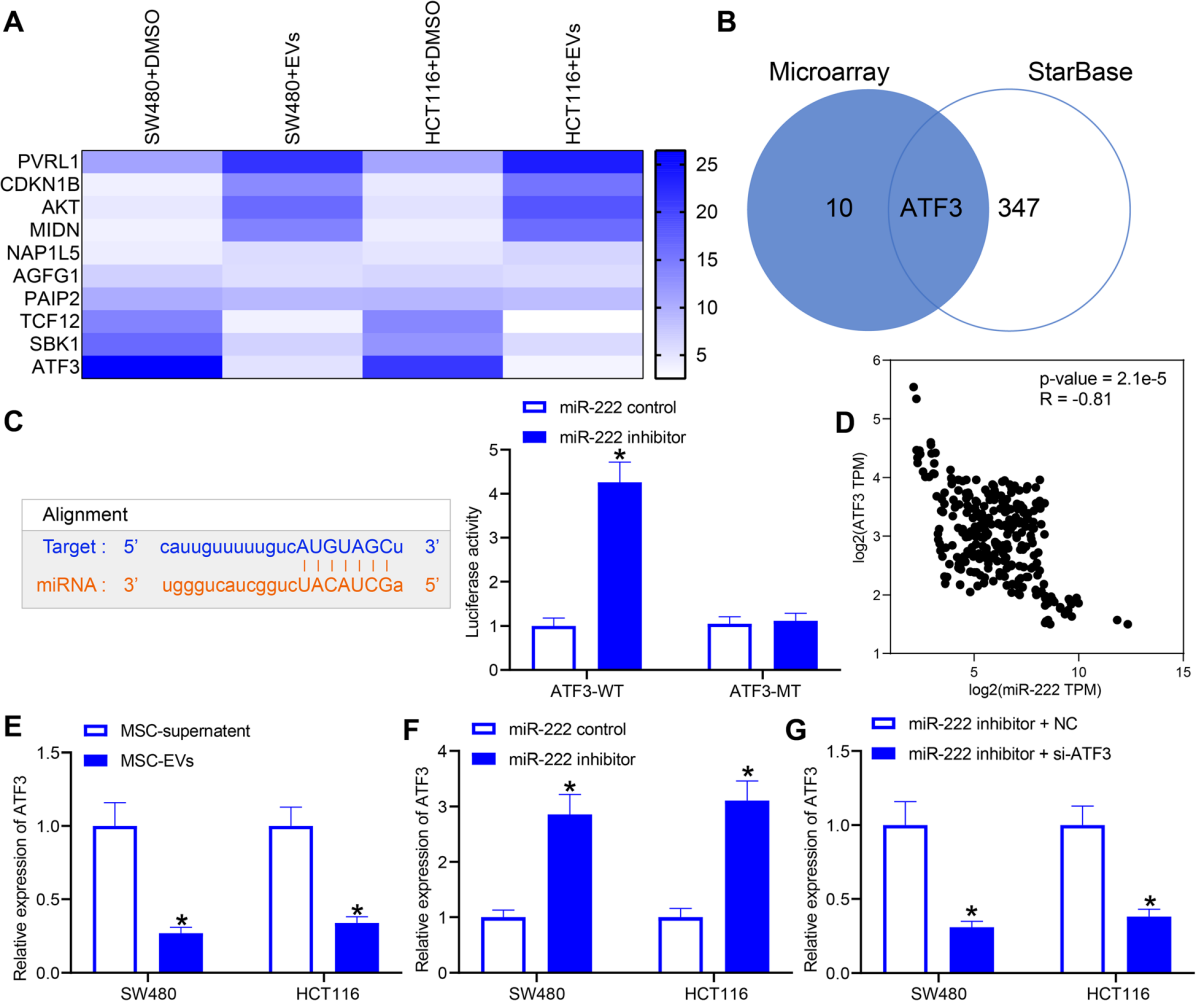
ATF3 is a downstream target of miR-222 in CRC cells. **a**, microarray analysis of mRNA expression changes in CRC cells before and after EVs treatment using R (Version 3.6.3, ww.r-project.org); **b**, the target genes of miR-222 in CRC depicted by Venn map; **c**, the targeting relationship between miR-222 and ATF3 verified by dual-luciferase assay; **d**, the association of miR-222 with ATF3 expression in CRC predicted by TCGA database; **e**, changes in ATF3 expression in CRC cells after co-culture determined by RT-qPCR; **f**, mRNA expression of ATF3 in cells measured by RT-qPCR; **g**, mRNA expression of ATF3 in cells after co-transfection analyzed by RT-qPCR. Two-way ANOVA was utilized to analyze data among multiple groups, followed by Tukey’s post hoc test

To test the effect of poor expression of miR-222 on CRC carcinogenesis and immune escape, we implanted EVs-treated SW480 cells with poor expression of miR-222 in mice. Tumor volume changes in mice was measured every 1 week and a tumor growth curve was thus plotted (Fig. 3a). Downregulation of miR-222 resulted in smaller growth volume and slower tumor growth in mice, possibly through ameliorating the effects of MSCs-EVs. Twenty-wight days later, measurement of mouse tumor weight revealed that downregulation of miR-222 reduced tumor formation (Fig. 3b). For a potential role of miR-222 in immunity, we resected mouse tumors and performed CD3 immunohistochemical staining to assess T-cell density in tumors (Fig. 3c). More CD3^+^ cells were found in the immunohistochemical image following miR-222 knockdown. Detection of miR-222 levels in tumors by in situ hybridization revealed a significant reduction in miR-222 levels in cells with higher T-cell density (Fig. 3d). Analysis of protein expression of human leukocyte antigen-A (HLA-A), apoptosis antigen 1 (Fas), c-c chemokine receptor type 5 (CCR5), Fas ligand (FasL) and HLA-E in tumor tissues demonstrated that in the tissues of mice with poor miR-222 expression, a significant increase in Fas protein expression and a significant decline in HLA-A, CCR5, FasL, and HLA-E protein expression were observed (Fig. 4e). The alteration of these proteins suggested enhanced immune surveillance and reduced immune escape.

### miR-222 targets ATF3 in CRC cells

To search for binding genes downstream of miR-222, we performed microarray analysis of differentially expressed mRNAs in EVs-treated cells (Fig. 4a). The analysis showed seven upregulated genes and three downregulated genes in EV-treated SW480 cells, so we intersected the prediction results of StarBase with the microarray results (Fig. 4b). In the screening, ATF3 was as found both a target gene of miR-222 and reduced after EVs treatment. Dual-luciferase experiments were utilized to validate the relationship between miR-222 and ATF3 (Fig. 4c). By examining the fluorescence intensity, we found that downregulation of miR-222 resulted in a significant increase in fluorescence intensity in cells transfected with ATF3-WT plasmids. The correlation between miR-222 and ATF3 was detected in The Cancer Genome Atlas (TCGA) database, and miR-222 was found to be negatively correlated with ATF3 expression in CRC (Fig. 4d). The quantification of ATF3 mRNA expression by RT-qPCR first verified that ATF3 was downregulated with EV induction (Fig. 4e), and we detected an increase in ATF3 expression in cells with miR-222 knockdown (Fig. 4f). Later, we further downregulated ATF3 expression in cells where miR-222 was knocked-down for subsequent studies (Fig. 4g).

### Silencing of ATF3 abrogates the effects of miR-222 inhibitor on CRC cells

**Fig. 5:**
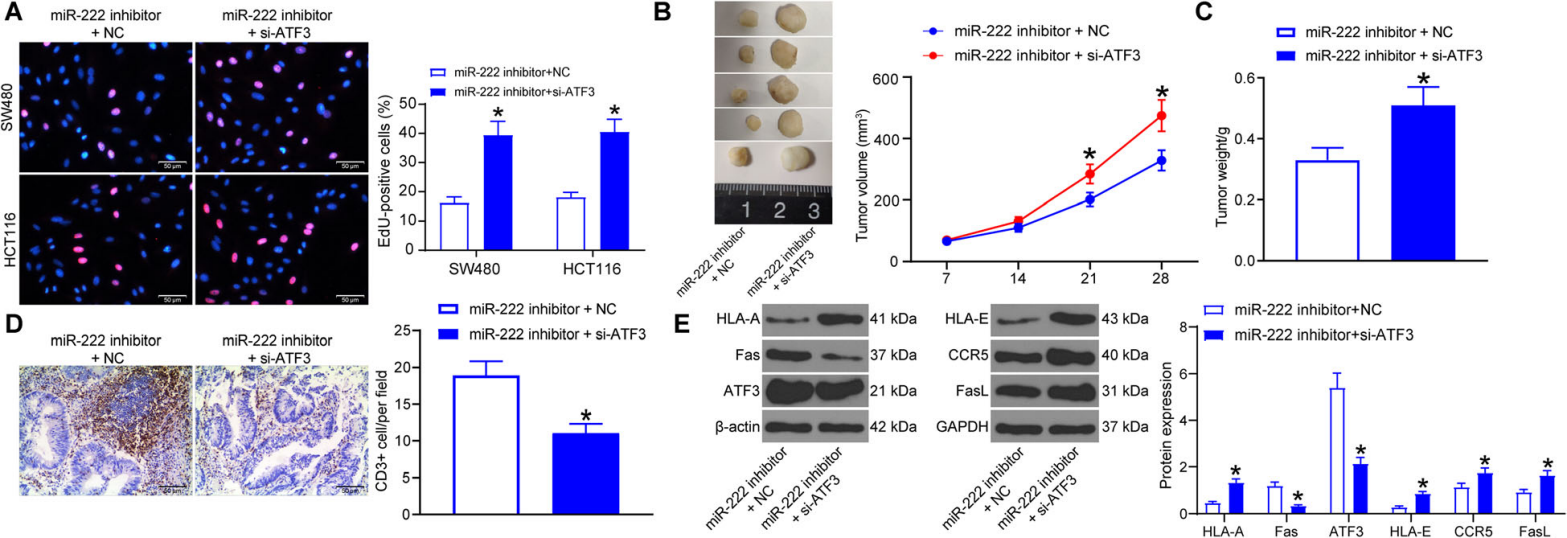
Silencing of ATF3 antagonizes the effects of miR-222 inhibitor on CRC cells. **a**, cell proliferation activity after inhibition of miR-222 and ATF3 assessed by EdU assay; **b**, tumor volume in xenograft models; **c**, assessment of tumorigenesis by tumor weight; **d**, percentage of CD3^+^ cells in tumor tissues detected by immunohistochemistry; **e**, expression of ATF3 and immune escape-related proteins in tumor tissues examined by western blot. Full-length blots are presented in Additional file 4 (Supplementary Fig. S3). Measurement data were presented as mean ± SD. Unpaired *t* test was applied to analyze the differences between two experimental groups (panel **c** and **d**), while two-way ANOVA was utilized to analyze data among multiple groups, along with Tukey’s post hoc test (panel **a** and **e**)

The combined effect of si-ATF3 and miR-222 inhibitor on the proliferation of CRC cells was assessed by EdU, which revealed a significant augment in the proliferation of CRC cells after ATF3 downregulation (Fig. 5a). Since the differences in the proliferative activity of SW480 cells were more pronounced, we implanted SW480 cells into mice to detect tumor formation and the immune microenvironment. The evaluation of mouse tumors revealed that downregulation of ATF3 partially restored tumorigenic capacity (Fig. 5b). Tumor weights of mice bearing miR-222 inhibitor + si-ATF3 were much heavier after 4 weeks than controls with poor expression of miR-222 alone (Fig. 5c). Immunohistochemistry for paraffin-embedded sections of mouse tumor tissues revealed reduced CD3^+^ cells and decreased T-cell density in the tissues (Fig. 5d). Moreover, we analyzed immunity-related molecules and ATF3 protein expression in tissues by western blot (Fig. 5e), and protein bands showed a significant decrease in Fas protein expression in tissues of mice poorly-expressing ATF3 and miR-222, while protein expression of HLA-A, CCR5, FasL and HLA-E was significantly increased. These results indicated that si-ATF3 led to enhanced tumor growth and immune escape in vivo.

### ATF3 negatively regulates AKT1 expression in CRC cells

**Fig. 6:**
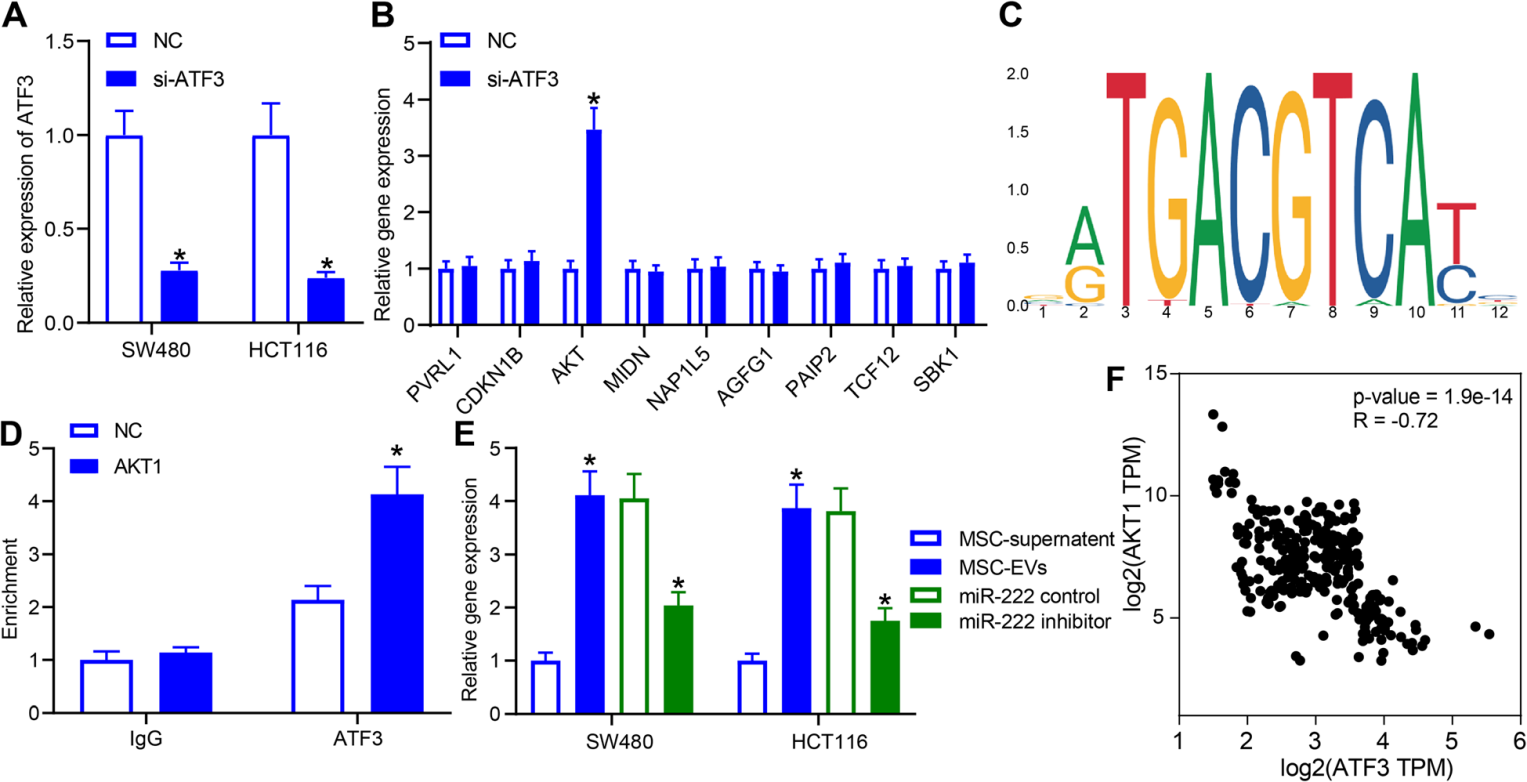
ATF3 interacts with AKT1 in CRC cells. **a**, ATF3 expression in CRC cells after ATF3 downregulation determined by RT-qPCR; **b**, quantification of genes in CRC cells after si-ATF3 assessed by RT-qPCR; **c**, possible binding sites for ATF3 and AKT1 predicted by JASPAR (http://jaspar.binf.ku.dk/); **d**, the binding of AKT1 and ATF3 validated by ChIP experiments; **e**, AKT1 expression in CRC cells after co-culture or transfection determined by RT-qPCR; **f**, the association of AKT1 with ATF3 expression in CRC predicted by TCGA database. Two-way ANOVA was utilized to analyze data among multiple groups, followed by Tukey’s post hoc test

Because ATF3 is a transcription factor that acts in the cell by regulating gene transcription, we think that ATF3 does not act directly on pathways, but mediates the expression of a gene. As a consequence, we knocked-down the expression of ATF3 in SW480 cells (Fig. 6a), and measured the mRNA expression of other nine genes that were dysregulated after EVs treatment in mRNA-based microarray analysis (Fig. 6b). AKT1 was found to be significantly upregulated in cells with ATF3 knockdown. The promoter sequence of AKT1 and the binding sites between ATF3 and AKT1 were obtained by bioinformatics analysis (Fig. 6c). By ChIP experiments, we found a binding relationship between ATF3 and AKT1 (Fig. 6d). RT-qPCR further displayed a significant AKT1 overexpression in EVs-treated cells, and a significant decrease in AKT1 expression in CRC cells co-cultured with MSCs-EVs and transfected with miR-222 inhibitor (Fig. 6e). A query of the expression correlation between AKT1 and ATF3 in the TCGA database revealed a negative correlation (Fig. 6f), indicating that AKT1 is an ATF3-regulated gene.

### ATF3 regulates the AKT pathway by binding to AKT1 in CRC cells

**Fig. 7:**
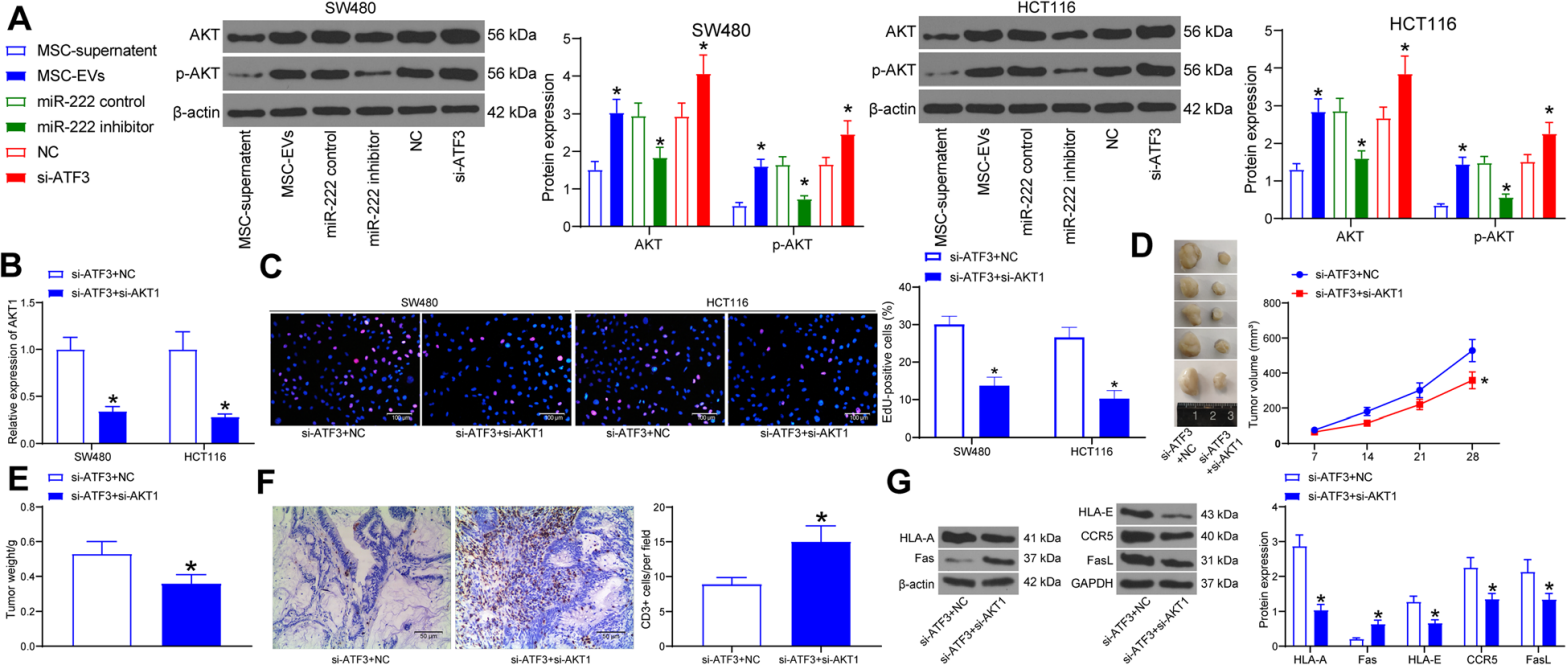
Silencing of AKT1 antagonizes the effects of ATF3 knockdown on CRC cells. **a**, the activation of the AKT pathway in CRC cells treated with MSC-EVs alone or with si-ATF3/miR-222 inhibitor determined by western blot. Full-length blots are presented in Additional file 5 (Supplementary Figure S4); **b**, AKT1 expression in CRC cells; **c**, cell proliferation activity after co-transfection in cells assessed by EdU assay; **d**, tumor volume in xenograft models; **e**, assessment of tumorigenesis by tumor weight; **f**, percentage of CD3^+^ cells in tumor tissues detected by immunohistochemistry; **g**, expression of immune escape-related proteins in tumor tissues examined by western blot. Full-length blots are presented in Additional file 6 (Supplementary Figure S5). Unpaired *t* test was applied to analyze the differences between two experimental groups (panel **f**), while two-way ANOVA was utilized to analyze data among multiple groups, along with Tukey’s post hoc test (panel **a**, **b**, **c**, **d**, **e** and **g**)

Since AKT1 is a key gene in the AKT pathway, we speculated whether ATF3 regulates the malignant phenotype of CRC cells by mediating the AKT pathway. AKT pathway activation was assessed by western blot in SW480 and HCT116 cells in response to differential gene expression. In EVs-treated cells, we found an increase in AKT pathway activation. miR-222 downregulation inhibited the action of MSCs-EVs, resulting in a significant decrease in AKT pathway activation in cells, while ATF3 downregulation contributed to the promoting effects of MSCs-EVs on the AKT pathway activation in cells (Fig. 7a). These evidences showed that the AKT pathway is the pathway mediated by ATF3 in CRC cells. Silencing of AKT1 was then delivered into CRC cells transfected with si-ATF3, and RT-qPCR verified the efficacy of co-transfection (Fig. 7b). AKT1 downregulation inhibited the effect of si-ATF3, resulted in significant decreases in proliferative activity of SW480 and HCT116 cells (Fig. 7c) and in tumorigenic activity of SW480 cells (Fig. 7d, e). At the same time, AKT1 downregulation increased CD3^+^ cells and T-cell density in tumor tissues (Fig. 7f). Furthermore, western blot showed that inhibition of AKT1 reversed the effect of si-ATF3 to significantly increase Fas protein expression in tumor tissues, while to significantly decrease HLA-A, CCR5, FasL, HLA-E protein expression (Fig. 7g), which implied that the immune escape of cancer cells was diminished.

## Discussion

Distinctive characteristics of MSCs make them highly promising in the cell-based therapy of cancers, and MSCs have the potency to suppress the immune system and support tumor cells to escape from immune responses [14]. On the other hand, tumor-derived EVs have been implicated in different events, including angiogenesis, chemoresistance as well as immune evasion, and their role has also been well-established in biological pathways involved in CRC initiation and progression [15]. In this study, we identified a novel regulatory mechanism expediting immune escape of CRC cells. We found that MSC produce EVs containing miR-222 to potentiate CRC cell malignant phenotype. miR-222 targets ATF3 in CRC cells and promotes immune escape of CRC cells by activating the AKT pathway.

The first finding of this study was that MSC-EVs encourage CRC cells to grow and proliferate. Ramírez-Ricardo et al. proposed that circulating EVs from patients with breast cancer could elevate migration and invasion of breast cancer cells [16]. In addition, tumor-derived EVs may contribute to favored tumor aggressiveness through both direct and indirect manners and are involved in tumor immune escape [17]. After that, we conducted microarray analysis to detect differential changes in miRNAs, followed by experimental validation. The upregulation of miR-222 in CRC cells was revealed as a result of MSC-EVs transfer. Further functional experiments were carried out in CRC cells with miR-222 knockdown. In addition to hampering malignant aggressiveness in vitro, miR-222 was also found to reduce tumor growth and immune escape in vivo. Consistent with our findings, endoplasmic reticulum stress-evoked exosomal miR-27a-3p promoted immune escape in breast cancer [18]. As regards to the function of miR-222, increased miR-222-3p expression was associated with metastasis and a poor prognosis in renal clear cell carcinoma [19]. In addition, miR-222 was enriched in retinoblastoma tissues and cells, which facilitated resistance of retinoblastoma cells to vincristine, a chemotherapeutic agent [20]. Under the context of CRC, HCT116 and SW480 cells illustrated repressed invasion and migration abilities and enhanced apoptosis in response to miR-222-3p inhibitor [21]. During the malignant transformation of normal colorectal epithelial cells, CCR5, FasL and HLA-E expression elevated remarkably, whereas Fas expression reduced [22]. By contrast, we observed that miR-222 inhibitor diminished HLA-A, CCR5, FasL and HLA-E expression, while restored Fas expression, implying that that the immune escape was prevented. Likewise, Cojo et al. believed that upregulation of hsa-miR-222 could protect against apoptosis in HIV-infected CD4^+^ T cells [23].

In this study, we also noted that miR-222 had a binding relationship with ATF3, and ATF3 was not only a target of miR-222, but also downregulated in CRC cells after MSC-EVs treatment. Interestingly, overexpression of ATF3 was linked to good survival rates in CRC patients, and silencing of ATF3 promoted proliferation, migration, and clonogenic growth of CRC cells [24]. Consistently, our rescue experiments disclosed that downregulation of ATF3 reversed the inhibitory effects of miR-222 knockdown on CRC cell growth and immune escape. Kim et al. reported that ATF3, a member of the ATF/CREB family of transcription factors, is tightly related to apoptosis in CRC cells with the involvement of many signaling components, including AKT [25]. Moreover, the depletion of ATF3 promoted activation of the AKT signaling, evidenced by higher extent of AKT phosphorylation, to accelerate prostate cancer development [26]. Our bioinformatics analysis and ChIP assays provided evidence that ATF3 directly bound to and shared a negative correlation with AKT1 in CRC cells. Further western blot analyses established that miR-222 inhibitor resulted in the AKT pathway deficit, while si-ATF3 led to the AKT pathway activation. Also, high-mobility group A1 expedited uveal melanoma progression via the PI3K/AKT pathway and oncogenic miR-222 [27]. In the same vein, tumor-secreted exosomal miR-222 facilitated pancreatic cancer progression by potentiating the AKT pathway [28]. Additional rescue experiments in our study indicated that AKT1 knockdown reversed the supporting role of si-ATF3 in tumor growth and immune escape in CRC.

## Conclusion

**Fig. 8:**
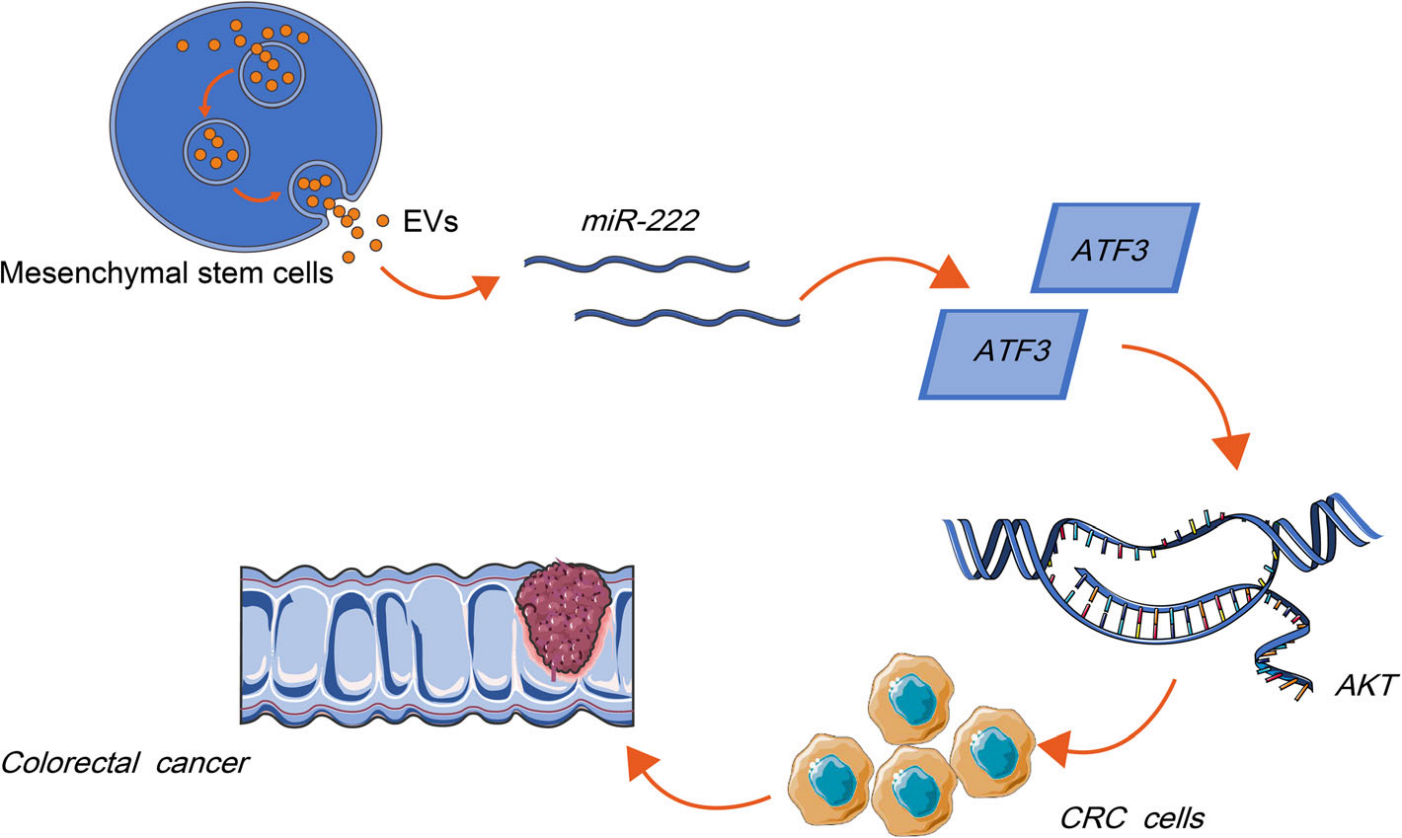
Mechanistic scheme depicting immune escape of CRC cells via MSC-EVs (Adobe Illustrator CS6, Adobe, San Jose, CA, USA). Secretion of MSC-EVs into CRC cells increased the expression of miR-222 in the cells. miR-222 targeted ATF3 to inhibit its activity, which led to the activation of ATF3-regulated AKT pathway and a subsequent increase in CRC cell malignant phenotype and immune escape

Altogether, our study proposed an interaction approach between CRC cells and MSC-EVs, in which miR-222 derived from MSC-EVs committed the posttranscriptional regulation on ATF3, consequentially activated the AKT pathway and promoted the development of CRC, thereby augmenting tumor growth and immune escape in vivo (Fig. 8). These data may offer novel insights for future CRC treatment options. However, whether the expression and function of miR-222 are affected by the CRC stage in clinic has yet to be established. Moreover, exosomes derived from human umbilical cord MSCs have been reported to relieve inflammatory bowel disease in mice [29]. It is well-known that inflammatory condition is another important process during the cancer progression in addition to immune escape. Therefore, figuring out the possible mechanism of MSC-EVs-derived miR-222 in inflammation could also be the next direction of our investigation. In addition, the lack of applying miR-222 inhibitor alone in CRC cells may be another limitation of our study. We believe that the resolving of these problems is helpful for clinical translation for MSC-EVs-derived miR-222.

## Supplementary Information

**Additional file 1: Supplementary Table S1** Antibodies used in this study.

**Additional file 2: Supplementary materials S1** and **Supplementary Figure S1** MSC-EVs are extracted and co-cultured with CRC cells.

**Additional file 3: Supplementary Figure S2** Full-length western blots of Fig. 3e.

**Additional file 4: Supplementary Figure S3** Full-length western blots of Fig. 5e.

**Additional file 5: Supplementary Figure S4** Full-length western blots of Fig. 7a.

**Additional file 6: Supplementary Figure S5** Full-length western blots of Fig. 7g.

**Additional file 7: Supplementary Figure S6** Full-length western blots of Figure S1E.

## Data Availability

All data generated or analysed during this study are included in this published article [and its supplementary information files].

## Abbreviations

CRC: Colorectal cancer
miR-222: Microrna-222
MSC-EVs: Mesenchymal stem cells-derived extracellular vesicles
ATF3: Activating transcription factor 3
PBS: Phosphate buffer saline
DMEM: Dulbecco’s modified Eagle’s medium
FBS: Fetal bovine serum
NTA: Nanoparticle tracking analysis
TEM: Transmission electron microscopy
TSG101: Tumor susceptibility gene 101
si: Small interfering RNA
EdU: 5-Ethynyl-2′-deoxyuridine
cDNA: Complementary DNA
RT-qPCR: Reverse transcriptase quantitative PCR
GAPDH: Glyceraldehyde-3-phosphate dehydrogenase
FITC: Fluorescein isothiocyanate
PI: Propidium iodide
WT: Wild-type
MT: Mutant
3’UTR: 3’untranslated region
ChIP: Chromatin immunoprecipitation
SDS: Sodium dodecyl sulfate
OD: Optical density
HLA-A: Human leukocyte antigen-A
Fas: Apoptosis antigen 1
CCR5: C-c chemokine receptor type 5
FasL: Fas ligand
