# Supplementary material for: Extracellular vesicles-derived microRNA-222 promotes immune escape via interacting with ATF3 to regulate AKT1 transcription in colorectal cancer
Source: BMC Cancer. 2021 Apr 1;21:349. doi: 10.1186/s12885-021-08063-5 (PMC8017736; doi:10.1186/s12885-021-08063-5)
Supplement: Supplementary file 1 — Additional file 1: Supplementary Table S1 Antibodies used in this study. [file 12885_2021_8063_MOESM1_ESM.docx]

**Supplementary Table S1** Antibodies used in this study

| Assays | Antibodies | Catalog number | Dilution | Manufacturer |
| --- | --- | --- | --- | --- |
| Western blot | TSG101 (mouse) | GTX70255 | 1:500 | GeneTex |
|  | CD81 (mouse) | sc-23962 | 1:500 | Santa Cruz Biotechnology |
|  | GM130  (mouse) | 66662-1-Ig | 1:1000 | Proteintech Group |
|  | HLA-A (rabbit) | ab52922 | 1:100 | Abcam |
|  | Fas (rabbit) | ab178076 | 1:300 | Abcam |
|  | ATF3 (mouse) | sc-188 | 1:500 | Santa Cruz Biotechnology |
|  | HLA-E (rabbit) | ab2216 | 1:700 | Abcam |
|  | CCR5 (rabbit) | MAB1802 | 1:1000 | R&D Systems |
|  | FasL (rabbit) | MAB126 | 1:300 | R&D Systems |
|  | AKT (mouse) | sc-8312 | 1:500 | Santa Cruz Biotechnology |
|  | p-AKT (rabbit) | ab81283 | 1:500 | Abcam |
|  | β-actin (mouse) | ab8226 | 1:300 | Abcam |
|  | GAPDH (rabbit) | #5174 | 1:1000 | Cell Signal Technology |
|  | Goat anti-rabbit secondary antibody | ab205718 | 1:5000 | Abcam |
|  | Goat anti-mouse secondary antibody | ab205719 | 1:5000 | Abcam |
| Immunohistochemistry | CD3 (rabbit) | ab16669 | 1:150 | Abcam |
|  | Goat anti-rabbit secondary antibody | ab205718 | 1:3000 | Abcam |
| ChIP | ATF3 (mouse) | sc-188 | 1:500 | Santa Cruz Biotechnology |
|  | IgG | ab172730 | 1:100 | Abcam |

**Note:** ChIP, chromatin immunoprecipitation.
