# Supplementary material for: Extracellular vesicles-derived microRNA-222 promotes immune escape via interacting with ATF3 to regulate AKT1 transcription in colorectal cancer
Source: BMC Cancer. 2021 Apr 1;21:349. doi: 10.1186/s12885-021-08063-5 (PMC8017736; doi:10.1186/s12885-021-08063-5)
Supplement: Supplementary file 2 — Additional file 2: Supplementary materials S1 and Supplementary Figure S1 MSC-EVs are extracted and co-cultured with CRC cells. [file 12885_2021_8063_MOESM2_ESM.docx]

**Supplementary materials S1**

**MSC-EVs induce CRC cell activity**

We cultured MSC-like cells with typical long spindle morphology from human CRC tissues under nutritional conditions and examined the cell morphology by microscopy (Supplementary Fig. S1A). The immunophenotypes of the cells were identified by flow cytometry to validate MSC-like cells, and the MSC-like cells were found positive for CD73 (98.6%), CD90 (99.8%) and CD105 (95.2%), while negative for CD14 (0.1%), CD19 (0.1%), CD45 (0.1%) (Supplementary Fig. S1B). EVs were separated from MSCs by ultra-centrifugation and the peak particle size of EVs was analyzed by NTA (Supplementary Fig. S1C). The peak particle size of natural extracellular vesicles from cell culture supernatants ranged from 35 to 184 nm, with an average particle size of 83.3 ± 24.8 nm. The peak distribution of EVs produced by standard separation techniques after ultracentrifugation ranged from 50 nm to 150 nm with an average value of 91 ± 18.3 nm. The morphology of the isolated EVs was studied by TEM (Supplementary Fig. S1D), and the ultrastructure of the EVs under TEM showed small oval-shaped membrane vesicles, conforming to the expected size distribution and possessing membrane integrity. The existence of markers TSG101 and CD81 and the absence of GM130 (Supplementary Fig. S1E) were detected in lysates by western blot, and we confirmed successful extraction of EVs. EVs were co-cultured with CRC cells SW480 and HCT116 to test whether the EVs have an effect on CRC cell activity. Changes in cellular activity were assessed by detecting the proportion of cells positive for EdU using the EdU assay (Supplementary Fig. S1F). Observations under fluorescence microscopy revealed that MSC-EVs treatment induced a notable increase in the cellular activity of CRC cells SW480 and HCT116, indicating that EVs isolated from cancer tissue-derived MSCs induced the activity of CRC cells, which provided direction for our subsequent experiments.

**Supplementary Figure S1**


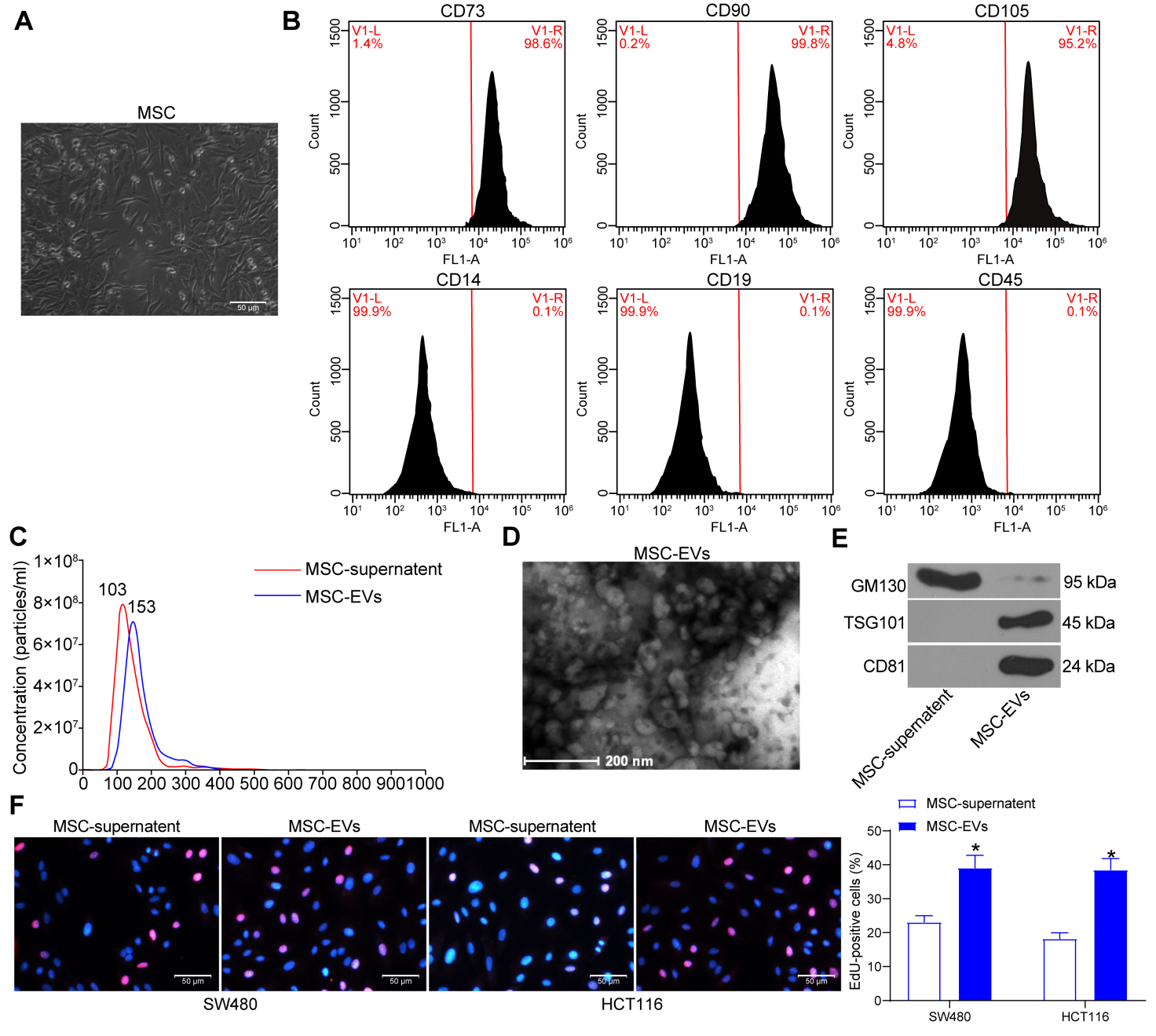


**Supplementary Figure S1** MSC-EVs are extracted and co-cultured with CRC cells. A, microscopic observation of MSC-like cell morphology; B, flow cytometry detection of cellular immunophenotypes to identify MSCs; C, peak particle size of EVs by NTA; D, morphological identification of EVs by TEM observation; E, the expression of the marker proteins TSG101, CD81, and GM130 (negative) assessed by western blot. Full-length blots are presented in Additional file 3 (Supplementary Figure S6); F, EdU assay for cell proliferation activity. Measurement data were exhibited as mean ± SD. Two-way ANOVA was utilized to analyze data among multiple groups, along with Tukey's post hoc test.
