# Supplementary figures and images for: Extracellular vesicles-derived microRNA-222 promotes immune escape via interacting with ATF3 to regulate AKT1 transcription in colorectal cancer
Source: BMC Cancer. 2021 Apr 1;21:349. doi: 10.1186/s12885-021-08063-5 (PMC8017736; doi:10.1186/s12885-021-08063-5)

**Supplementary Figure S2** Full-length western blots of Figure 3E.


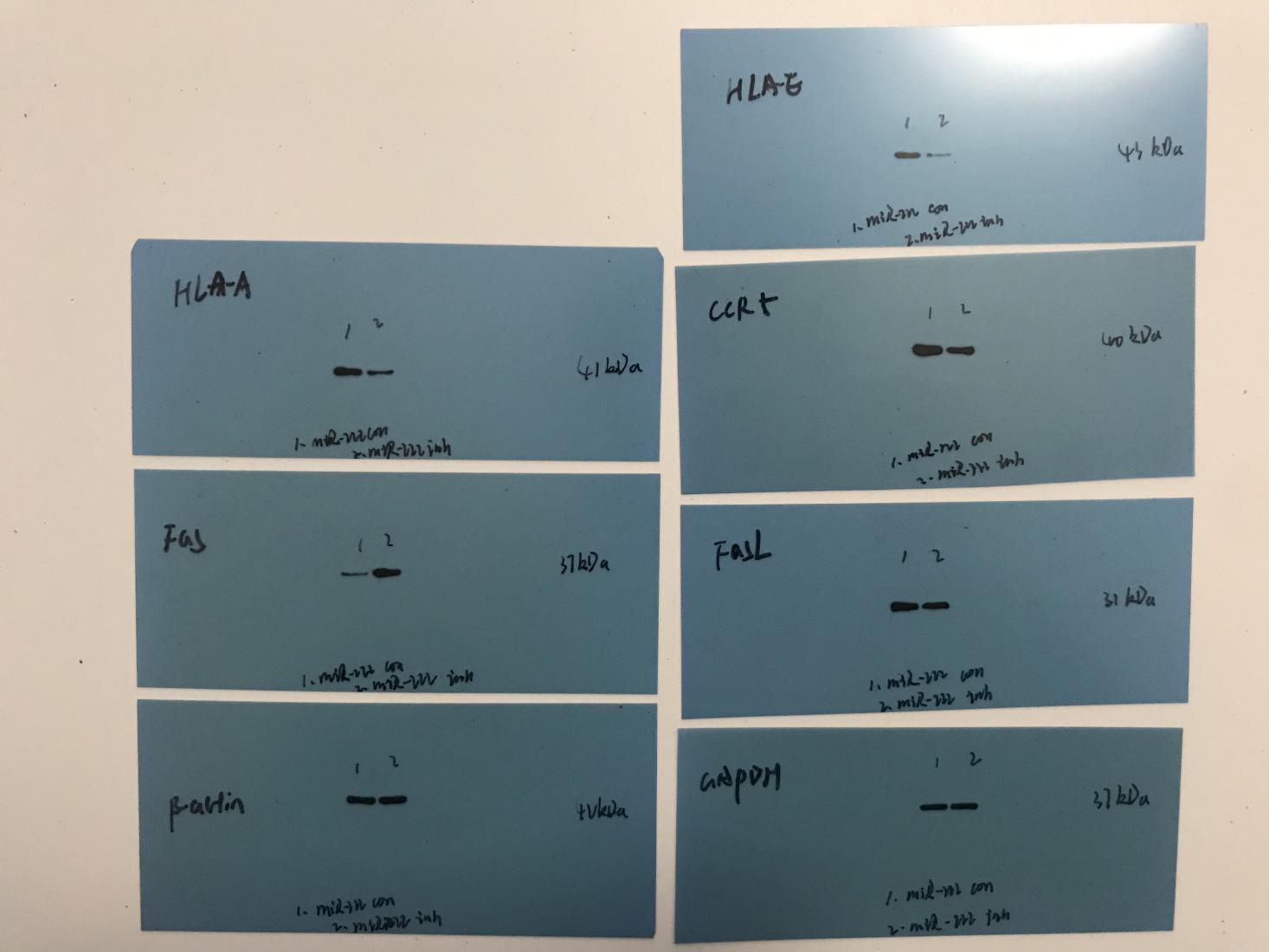

Supplement: Supplementary file 3 — Additional file 3: Supplementary Figure S2 Full-length western blots of Fig. 3e. [file 12885_2021_8063_MOESM3_ESM.docx]

**Supplementary Figure S4** Full-length western blots of Figure 5E.


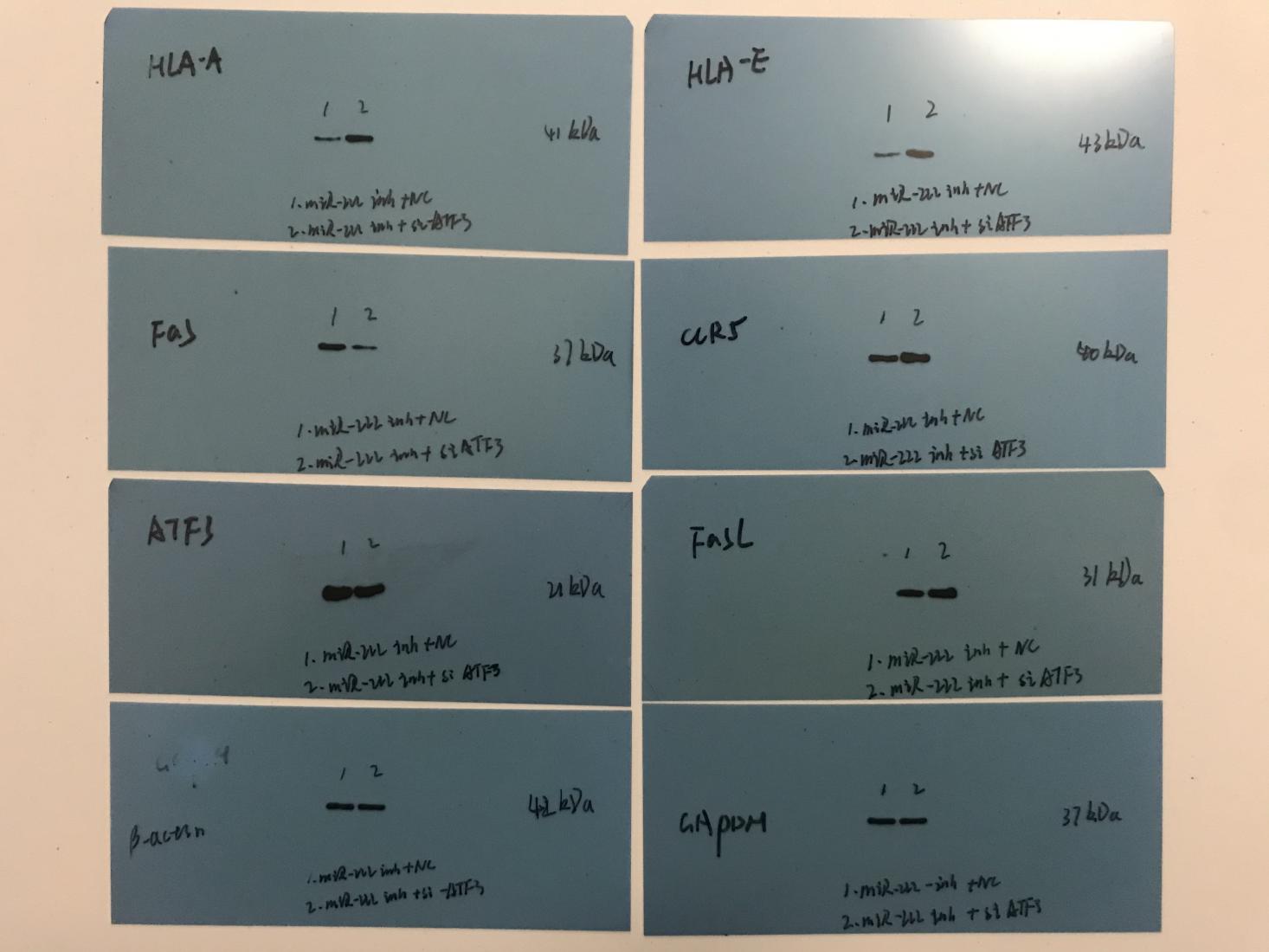

Supplement: Supplementary file 4 — Additional file 4: Supplementary Figure S3 Full-length western blots of Fig. 5e. [file 12885_2021_8063_MOESM4_ESM.docx]

**Supplementary Figure S4** Full-length western blots of Figure 7A.


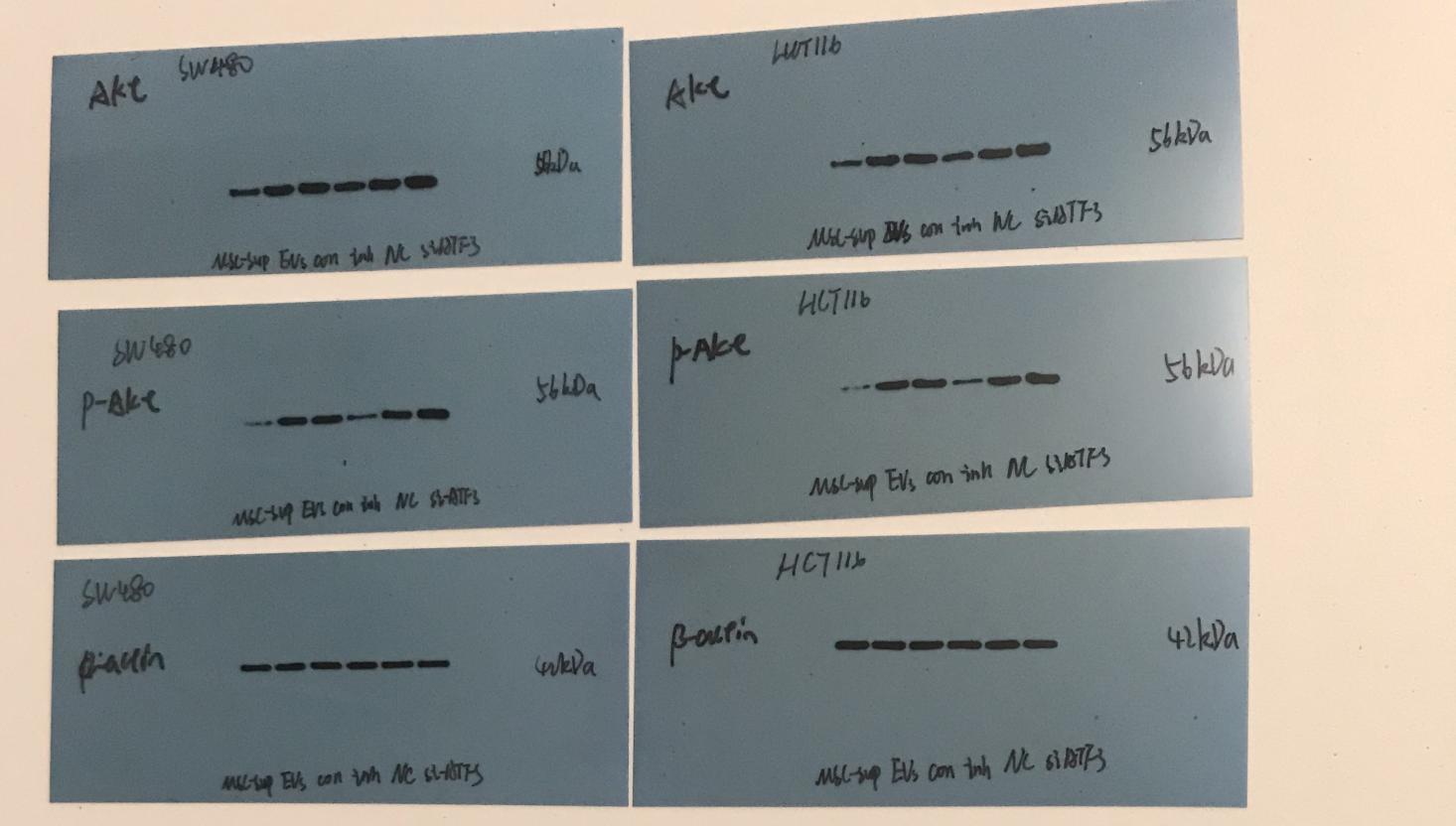

Supplement: Supplementary file 5 — Additional file 5: Supplementary Figure S4 Full-length western blots of Fig. 7a. [file 12885_2021_8063_MOESM5_ESM.docx]

**Supplementary Figure S5** Full-length western blots of Figure 7G.


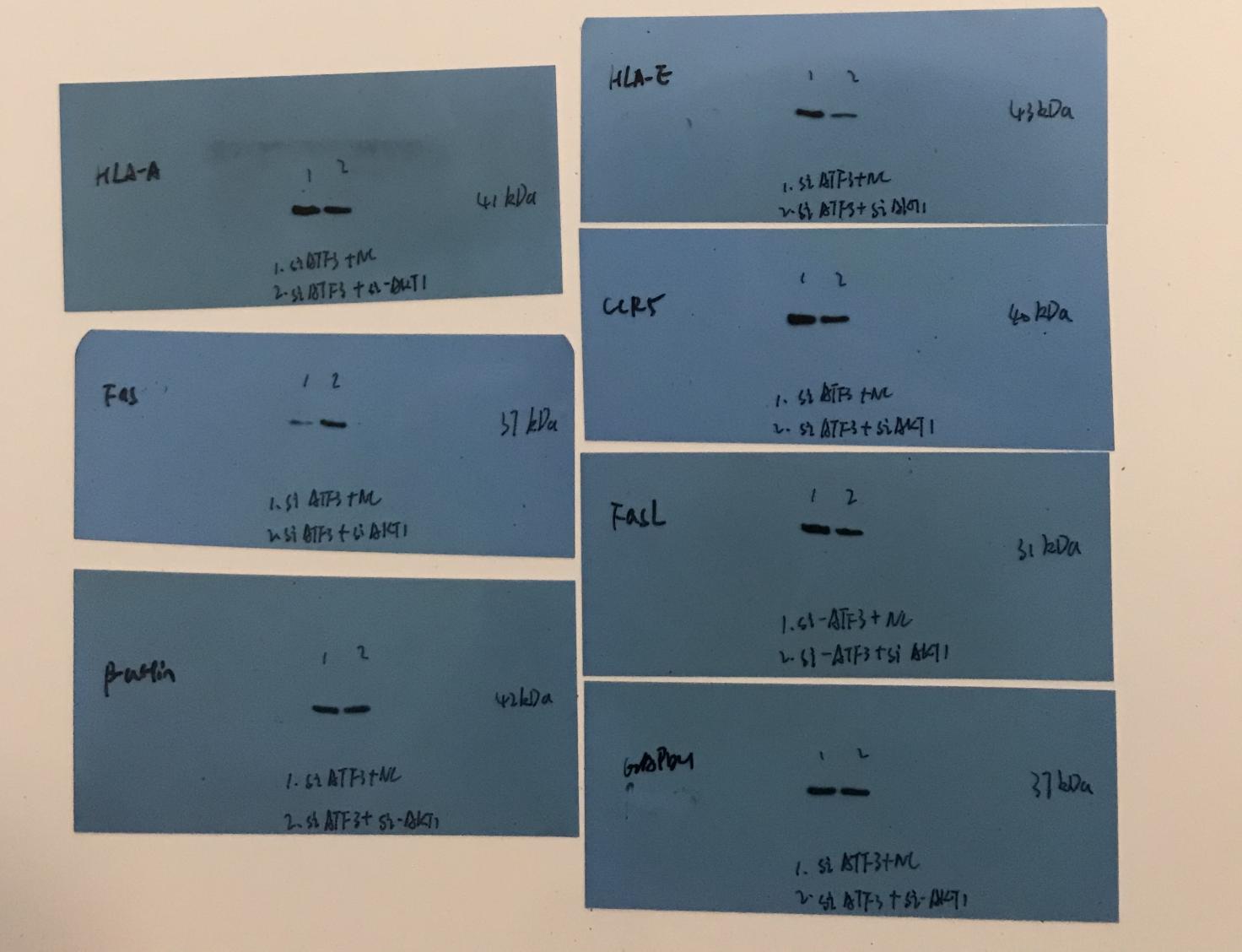

Supplement: Supplementary file 6 — Additional file 6: Supplementary Figure S5 Full-length western blots of Fig. 7g. [file 12885_2021_8063_MOESM6_ESM.docx]

**Supplementary Figure S6** Full-length western blots of Figure S1E.


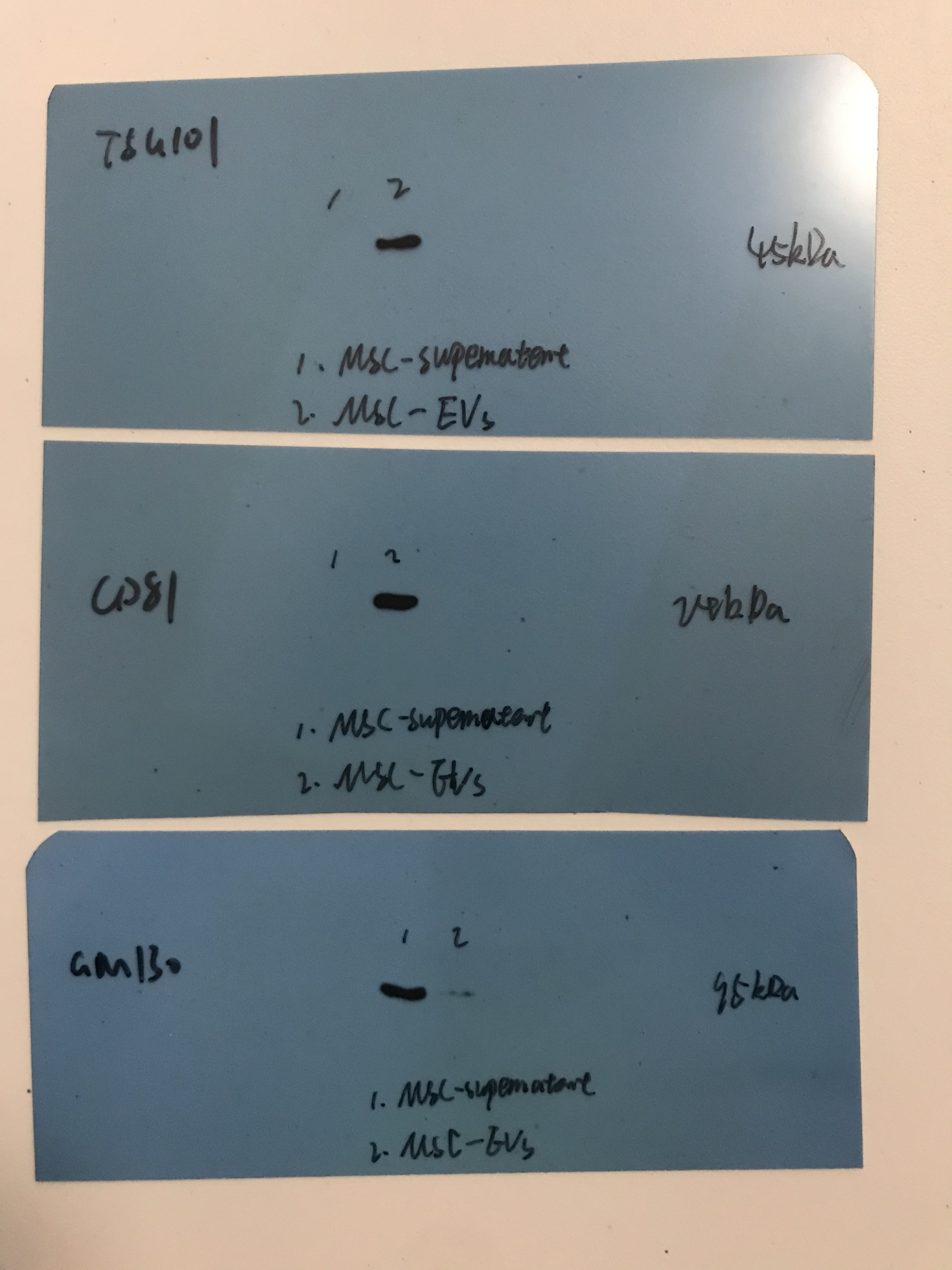

Supplement: Supplementary file 7 — Additional file 7: Supplementary Figure S6 Full-length western blots of Figure S1E. [file 12885_2021_8063_MOESM7_ESM.docx]
